# Supplementary material for: Causal Relationship between Aging and Anorexia Nervosa: A White-Matter-Microstructure-Mediated Mendelian Randomization Analysis
Source: Biomedicines. 2024 Aug 16;12(8):1874. doi: 10.3390/biomedicines12081874 (PMC11351342; doi:10.3390/biomedicines12081874)
Supplement: Supplementary file 1 [file biomedicines-12-01874-s001.zip › Supplementary Figure/Figure S4/Singe SNP plot of MR analysis for TL to ubm-b-1789.pdf]

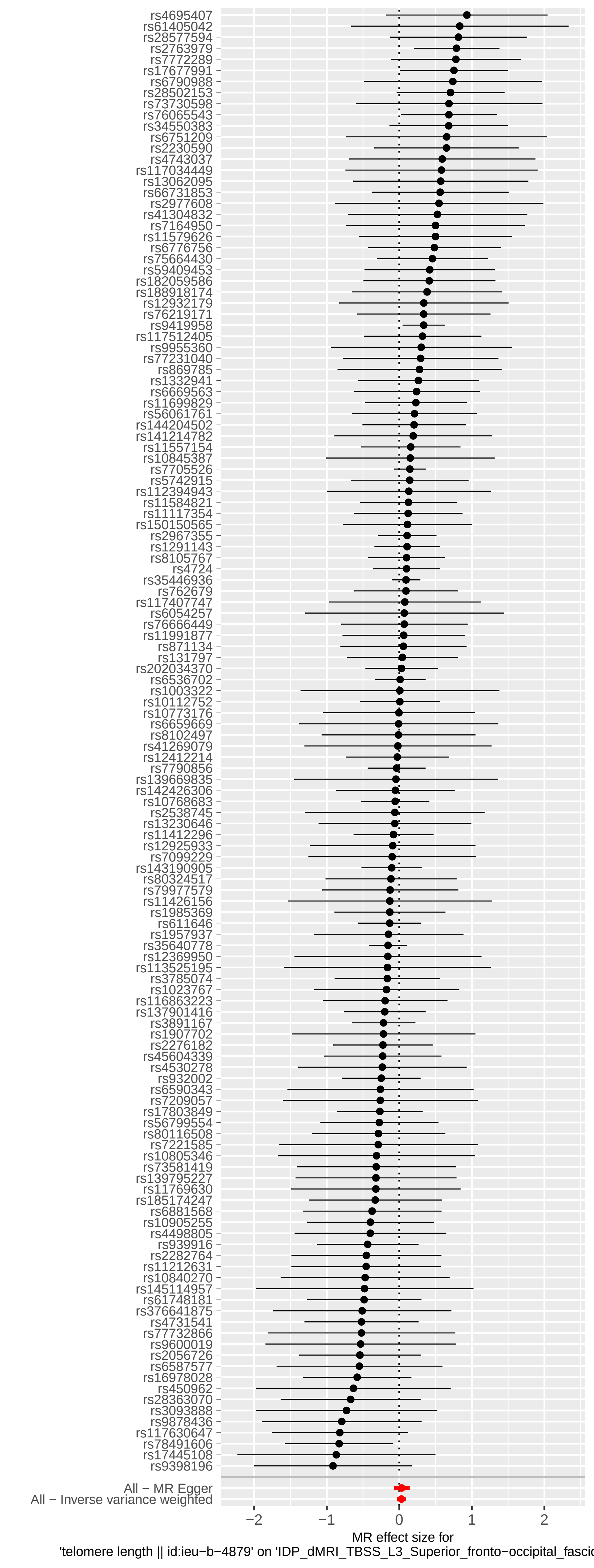
rs4695407
rs61405042
rs28577594
rs2763979
rs7772289
rs17677991
rs6790988
rs28502153
rs73730598
rs76065543
rs34550383
rs6751209
rs2230590
rs4743037
rs117034449
rs13062095
rs66731853
rs2977608
rs41304832
rs7164950
rs11579626
rs6776756
rs75664430
rs59409453
rs182059586
rs188918174
rs12932179
rs76219171
rs9419958
rs117512405
rs9955360
rs77231040
rs869785
rs1332941
rs6669563
rs11699829
rs56061761
rs144204502
rs141214782
rs11557154
rs10845387
rs7705526
rs5742915
rs112394943
rs11584821
rs11117354
rs150150565
rs2967355
rs1291143
rs8105767
rs4724
rs35446936
rs762679
rs117407747
rs6054257
rs76666449
rs11991877
rs871134
rs131797
rs202034370
rs6536702
rs1003322
rs10112752
rs10773176
rs6659669
rs8102497
rs41269079
rs12412214
rs7790856
rs139669835
rs142426306
rs10768683
rs2538745
rs13230646
rs11412296
rs12925933
rs7099229
rs143190905
rs80324517
rs79977579
rs11426156
rs1985369
rs611646
rs1957937
rs35640778
rs12369950
rs113525195
rs3785074
rs1023767
rs116863223
rs137901416
rs3891167
rs1907702
rs2276182
rs45604339
rs4530278
rs932002
rs6590343
rs7209057
rs17803849
rs56799554
rs80116508
rs7221585
rs10805346
rs73581419
rs139795227
rs11769630
rs185174247
rs6881568
rs10905255
rs4498805
rs939916
rs2282764
rs11212631
rs10840270
rs145114957
rs61748181
rs376641875
rs4731541
rs77732866
rs9600019
rs2056726
rs6587577
rs16978028
rs450962
rs28363070
rs3093888
rs9878436
rs117630647
rs78491606
rs17445108
rs9398196
All − MR Egger
All − Inverse variance weighted
−2
−1
0
1
2
MR effect size for
'telomere length || id:ieu−b−4879' on 'IDP_dMRI_TBSS_L3_Superior_fronto−occipital_fascic
